# Synaptotagmins 3 and 7 mediate the majority of asynchronous release from synapses in the cerebellum and hippocampus

**DOI:** 10.1016/j.celrep.2024.114595

**Published:** 2024-08-06

**Authors:** Dennis J. Weingarten, Amita Shrestha, Daniel J. Orlin, Chloé L. Le Moing, Luke A. Borchardt, Skyler L. Jackman

**Affiliations:** 1 Vollum Institute, Oregon Health & Science University, Portland, OR, USA; 2 These authors contributed equally; 3 Lead contact

## Abstract

Neurotransmitter release consists of rapid synchronous release followed by longer-lasting asynchronous release (AR). Although the presynaptic proteins that trigger synchronous release are well understood, the mechanisms for AR remain unclear. AR is sustained by low concentrations of intracellular Ca^2+^ and Sr^2+^, suggesting the involvement of sensors with high affinities for both ions. Synaptotagmin 7 (SYT7) partly mediates AR, but substantial AR persists in the absence of SYT7. The closely related SYT3 binds Ca^2+^ and Sr^2+^ with high affinity, making it a promising candidate to mediate AR. Here, we use knockout mice to study the contribution of SYT3 and SYT7 to AR at cerebellar and hippocampal synapses. AR is dramatically reduced when both isoforms are absent, which alters the number and timing of postsynaptic action potentials. Our results confirm the long-standing prediction that SYT3 mediates AR and show that SYT3 and SYT7 act as dominant mechanisms for AR at three central synapses.

## In brief

Weingarten et al. show that asynchronous neurotransmitter release prolongs the temporal window for postsynaptic firing in the cerebellum and hippocampus. Genetic deletion of synaptotagmin 3 and synaptotagmin 7 abolishes most asynchronous release, reducing information transfer between neurons.

## Graphical abstract

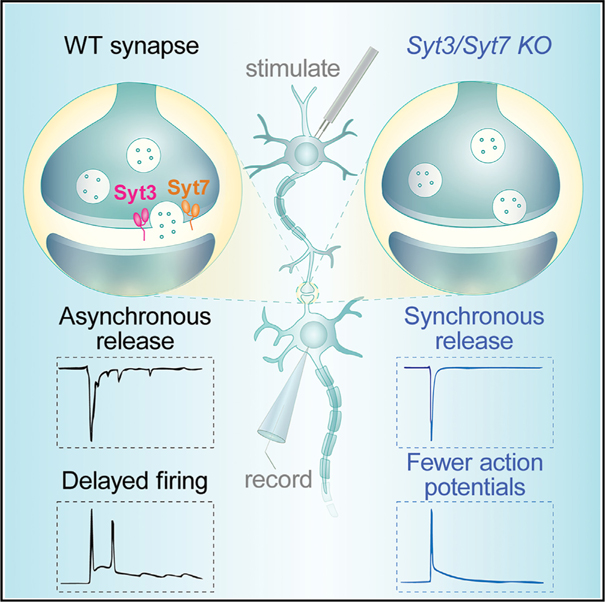

## INTRODUCTION

Presynaptic voltage-gated Ca^2+^ channels open during action potentials, briefly creating high local concentrations of Ca^2+^ near vesicle release sites (>10 μM) that trigger synchronous vesicle fusion with sub-millisecond precision.^1,2^ After each action potential, cytosolic Ca^2+^ drops to sub-micromolar “residual” concentrations and promotes lower rates of asynchronous release (AR) for tens to hundreds of milliseconds.^3^ For decades, synchronous release and AR were attributed to a single presynaptic Ca^2+^ sensor with steeply non-linear binding properties.^4^ However, after synaptotagmin 1 (SYT1) was identified as the Ca^2+^ sensor for vesicle fusion, genetic depletion of SYT1 was found to abolish synchronous release without affecting AR. This led to a “two-sensor” theory of vesicle fusion, where SYT1 promotes synchronous release while a second sensor promotes AR.^5,6^ Even though AR comprises a small fraction of total release at many synapses, its existence points to unidentified Ca^2+^ sensors that could regulate synaptic transmission and plasticity. Moreover, release at some synapses is almost entirely asynchronous,^7^ indicating that AR is developmentally regulated to perform specific functions in neural circuits.

Residual Ca^2+^ promotes AR and also several forms of short-term plasticity that increase synchronous release during high-frequency activity.^8^ AR and short-term plasticity exhibit similar time courses and Ca^2+^ dependencies, suggesting a common molecular basis.^5,9,10^ This long-standing hypothesis is supported by the recent identification of mechanisms that contribute to both phenomena.^11,12^ The best studied is SYT7, a high-affinity Ca^2+^ sensor involved in AR,^13^ vesicle replenishment,^14^ and short-term facilitation.^15^ However, genetic depletion of SYT7 only reduces AR by ~50%. Identifying the other sensors for AR would enable genetic manipulations to test AR’s hypothesized roles in shaping the timing and fidelity of postsynaptic firing^17,18^ and prolonging inhibition to suppress epileptiform activity.^19^

Here, we tested whether SYT3 contributes to AR at three central mammalian synapses. At cerebellar climbing fiber, which contain SYT3 but not SYT7, depleting SYT3 abolished the majority of AR. At cerebellar parallel fibers and synapses in the hippocampus between CA1 pyramidal cells and interneurons, knockout (KO) of either isoform reduced AR by half, and depleting both isoforms severely diminished AR. Synaptic responses in KO animals drove fewer action potentials in postsynaptic neurons, suggesting that AR driven by SYT3 and SYT7 can affect information transfer between neurons. AR from *Syt3* KO synapses showed almost no increase in the presence of Sr^2+^, suggesting SYT3 is responsible for the long-studied phenomena of Sr^2+^-induced AR. These findings establish SYT3 and SYT7 as potent drivers of AR, supporting the hypothesized link between AR and short-term plasticity.

## RESULTS

### SYT3 is critical for AR from cerebellar climbing fibers

**Figure 1.**
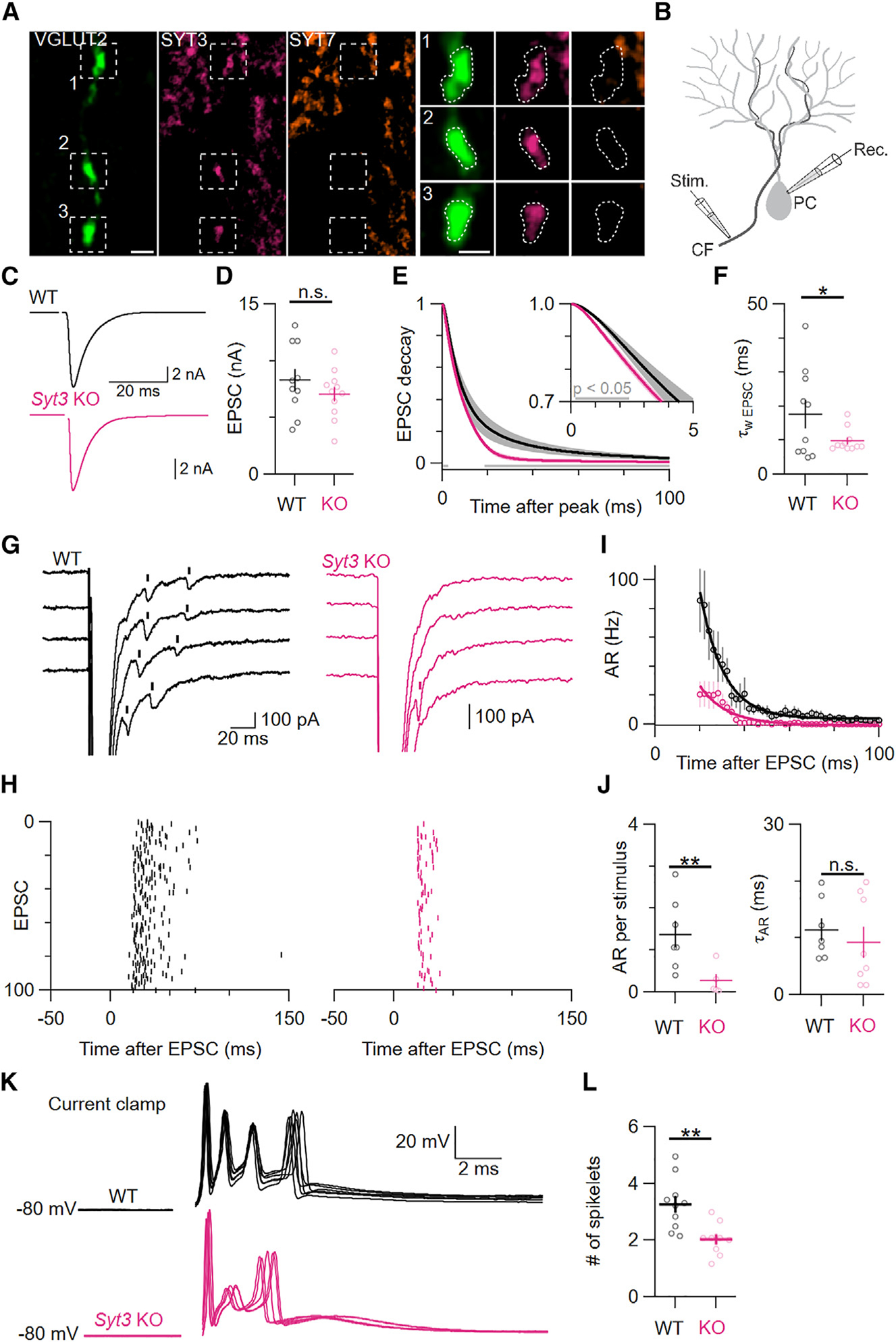
SYT3 is required for the majority of AR from CFs (A) High-magnification structured illumination microscopy (SIM) images of immunolabeling for VGLUT2 (green), SYT3 (magenta), and SYT7 (orange) in the cerebellar molecular layer and magnifications showing individual VGLUT2-positive boutons. Left scale bar: 2 μm; right scale bar: 1 μm. (B) Schematic for electrophysiological recordings of climbing fiber (CF) EPSCs in Purkinje cells (PCs). (C) Representative average of 100 EPSCs elicited by CF stimulation at 0.125 Hz in WT (black) and *Syt3* KO (magenta) synapses. (D) Peak EPSC amplitudes (*p* = 0.58). (E) Average decaying phase of EPSCs aligned to the peak with an inset showing the first 5 ms. Gray bars indicate significant differences between genotypes. (F) Weighted decay time constants of EPSCs (*p* = 0.03). (G) Representative synaptic responses in individual trials. Detected AR events are indicated by vertical bars above the raw traces (for the detection algorithm, see the STAR Methods). (H) Raster plots of detected AR events for 100 consecutive trials. (I) Average rate of AR after an EPSC in WT and *Syt3* KO synapses. Data were fit with single exponentials. (J) Quantification of AR events per stimulus (*p* = 0.007) and exponential time constant of AR (*p* = 0.26). (K) Five superimposed complex spikes evoked via CF stimulation at 0.125 Hz recorded from WT and *Syt3* KO cells current clamped to −80 mV. (L) Average number of spikelets per complex spike elicited by CF stimulation (*p* = 0.04). Averages shown with error bars represent mean ± SEM. Normal distribution was verified with Shapiro-Wilk tests. Subsequently, statistical significances were evaluated using two-tailed Student’s t tests. The number of experiments is shown in Table S1.

Cerebellar climbing fibers generate powerful synchronous excitatory postsynaptic currents (EPSCs) in Purkinje cells, followed by AR that can be detected as delayed quantal events.^20^ To determine whether SYT3 and SYT7 are present at these synapses, we performed immunolabeling for the vesicular glutamate transporter VGLUT2, which labels climbing fibers but not adjacent parallel fibers. As previously reported, climbing fibers were labeled by antibodies against SYT3, but not SYT7,^21,22^ making climbing fibers useful to study AR in the absence of SYT7 (Figure 1A). EPSCs were recorded from voltage-clamped Purkinje cells while stimulating climbing fiber axons at low frequency (0.125 Hz) in sagittal slices from wild-type (WT) and *Syt3* KO animals (Figure 1B). Synchronous EPSC amplitudes were similar in both genotypes (Figures 1C and 1D). Weighted time constants from bi-exponential curve fits revealed faster EPSC decay kinetics in *Syt3* KOs. Peak-aligned, averaged EPSC waveforms from *Syt3* KOs showed a subtle but significant decrease in amplitudes within <3 ms of the peak and at later time points (Figures 1E and 1F). We next used an automated algorithm to detect AR as delayed quantal events (see STAR Methods). The number of AR events following each stimulus was decreased by 80% in *Syt3* KOs, while the time course of the remaining AR did not change significantly (Figures 1G–1J). The amplitudes and kinetics of asynchronous events did not differ between genotypes (Figures S1A–S1C), suggesting that the reduction in AR could not be accounted for by changes in quantal size or altered density of postsynaptic AMPA receptors in KO animals. Thus, SYT3 promotes AR at synapses that do not express SYT7.

To test whether SYT3-driven AR affects postsynaptic firing, we performed current-clamp recordings from Purkinje cells while stimulating climbing fiber inputs. Climbing fiber EPSCs are so large that they cause Purkinje cells to fire brief, high-frequency discharges known as complex spikes. AR may influence the number of spikelets within complex spikes^23^ and affect the ability of spikelets to propagate down Purkinje cell axons and contribute to downstream information transfer.^18^ The number of spikelets also determines the amplitude of postsynaptic Ca^2+^ signals, thereby affecting cerebellar plasticity and motor learning.^24,25^ When we stimulated climbing fiber inputs while current clamping Purkinje cells at −80 mV, we found that *Syt3* KOs showed a 38% reduction in the number of spikelets driven by climbing fiber stimulation (Figures 1K and 1L). This change in complex spikes was not the result of altered postsynaptic excitability, as Purkinje cells in KOs showed similar spontaneous firing rates and excitability in response to current injections (Figure S2). These results demonstrate that SYT3-driven AR affects the firing of postsynaptic neurons.

### SYT3 and SYT7 are required for the majority of AR from cerebellar parallel fibers

**Figure 2.**
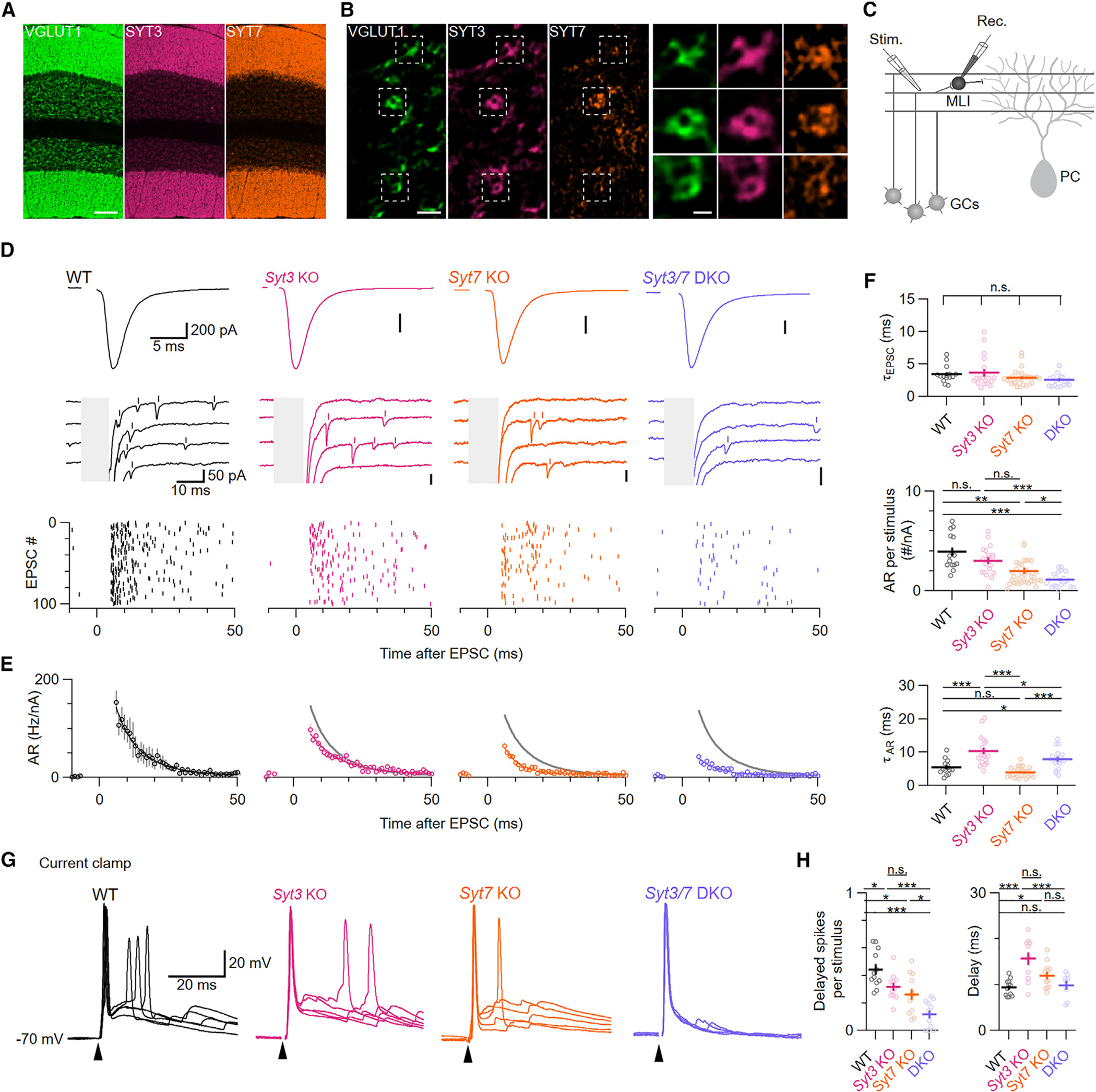
SYT3 and −7 are required for the majority of AR from parallel fibers (A) Immunolabeling for VGLUT1 (green), SYT3 (magenta), and SYT7 (orange) in the cerebellum. Scale bar: 100 μm. (B) High-magnification SIM images of immunolabeling for VGLUT1 (green), SYT3 (magenta), and SYT7 (orange) in the cerebellar molecular layer and zoom-ins of individual VGLUT1-positive parallel fiber boutons. Left scale bar: 2 μm; right scale bar: 500 nm. (C) Schematic for recording parallel fiber-to-MLI EPSCs. MLI, molecular layer interneuron; GCs, granule cells; PC, Purkinje cell. (D) Representative averages of 100 EPSCs elicited by parallel fiber stimulation with stimulation artifacts blanked (top). Examples of individual synaptic responses with synchronous EPSCs blanked (center). Detected AR events are indicated with vertical bars. Raster plots of detected AR and spontaneous events for 100 consecutive trials (bottom). (E) Time course of AR normalized to the number of activated parallel fibers (see the STAR Methods). Gray lines show fit to WT data for comparison. (F) Decay time constant of EPSCs (top; *p* = 0.14), normalized number of AR events per stimulus (center; WT vs. *Syt3* KO: *p* = 0.11; WT vs. *Syt7* KO: *p* = 0.0026; WT vs. DKO: *p* = 2.89 × 10^−7^; *Syt3* KO vs. *Syt7* KO: *p* = 0.05; *Syt3* KO vs. DKO: *p* = 7.50 × 10^−6^; *Syt7* KO vs. DKO: *p* = 0.01; critical a: 0.0167), and decay time constant of AR (bottom; WT vs. *Syt3* KO: *p* = 6.57 × 10^−5^; WT vs. *Syt7* KO: *p* = 0.03; WT vs. DKO: *p* = 0.01; *Syt3* KO vs. *Syt7* KO: *p* = 3.62 × 10^−8^; *Syt3* KO vs. DKO: *p* = 0.01; *Syt7* KO vs. DKO: *p* = 1.12 × 10^−6^; critical α: 0.0167). (G) Five superimposed examples of individual action potentials (APs) elicited by parallel fiber stimulation in current clamp at 0.125 Hz with stimulation artifacts blanked and indicated as arrowheads for clarity. Stimulation intensity was adjusted in voltage clamp to produce EPSC amplitudes of ~700 pA (WT: 716 ± 56 pA; Syt3 KO: 743 ± 63 pA; Syt7 KO: 727 ± 26 pA; DKO: 784 ± 42 pA; *p* = 0.87; critical a: 0.0167). (H) Number of delayed APs per stimulus (left; WT vs. *Syt3* KO: *p* = 0.01; WT vs. *Syt7* KO: *p* = 0.004; WT vs. DKO: *p* = 1.156 × 10^−6^; *Syt3* KO vs. *Syt7* KO: *p* = 0.28; *Syt3* KO vs. DKO: *p* = 1.28 × 10^−5^; *Syt7* KO vs. DKO: *p* = 0.01; critical α: 0.0167) and average delay of subsequent APs (right; WT vs. *Syt3* KO: *p* = 1.89 × 10^−5^; WT vs. *Syt7* KO: *p* = 0.012; WT vs. DKO: *p* = 0.62; *Syt3* KO vs. *Syt7* KO: *p* = 0.28; *Syt3* KO vs. DKO: *p* = 5.00 × 10^−5^; *Syt7* KO vs. DKO: *p* = 0.11; critical α: 0.0167). Averages shown with error bars represent mean ± SEM. Normal distribution was verified with Shapiro-Wilk tests. Subsequently, statistical significances were evaluated using ANOVA, followed by two-tailed Student’s t tests and a post hoc Šidák correction for multiple comparisons where necessary. The number of experiments is shown in Table S1.

Cerebellar parallel fibers are the other main glutamatergic synapses in the cerebellar cortex. Immunohistochemistry showed that SYT3 and SYT7 are both present in parallel fiber terminals, which can be distinguished from climbing fibers by labeling for VGLUT1 (Figures 2A and 2B). Due to the high density of synapses in the molecular layer, we could not easily distinguish individual terminals to calculate the proportion that stained for both SYT3 and SYT7. Therefore, as an alternative method to assess the degree of *Syt3* and *Syt7* co-expression, we analyzed single-nucleus RNA sequencing (snRNA-seq) data from granule cells, the neurons that give rise to parallel fibers. Using published snRNA-seq data from ~477,000 granule cell nuclei,^26^ we quantified the number of cells that expressed either isoform (Figure S3). Transcript detection errors guarantee that *Syt3* or *Syt7* RNA is not seen in all nuclei, and total transcript counts ranged by more than an order of magnitude. We therefore sorted nuclei by transcript counts and observed a strong correlation between transcript counts and the probability of detecting *Syt3* and *Syt7*. Co-detection of *Syt3* and *Syt7* occurred in 73% of nuclei with the most transcripts. The proportion of nuclei with only one isoform could be explained statistically by transcript detection errors. As a control, we repeated this analysis using snRNA-seq data from Purkinje cells, whose synaptic terminals stain for antibodies against SYT7^27^ but not SYT3.^21^ Purkinje cell snRNA-seq libraries differed markedly from granule cells: 97% of Purkinje cell nuclei expressed *Syt7* RNA, and only 5% were positive for *Syt3* and *Syt7*. In summary, immunohistochemistry and RNA expression suggest that *Syt3* and *Syt7* are widely co-expressed at the level of individual granule cells.

To evaluate the contribution of both isoforms to AR, we crossed *Syt3* KO and *Syt7* KO mice to generate constitutive *Syt3/Syt7* double-KO (DKO) animals. Immunolabeling for both SYT3 and SYT7 was absent in DKOs (Figure S4). We measured AR in all genotypes by recording from molecular layer interneurons (MLIs) while stimulating parallel fiber axons in transverse cerebellar slices (Figure 2C). Because MLIs receive input from many parallel fibers, we normalized AR rates to the size of the synchronous EPSC as a proxy for the number of stimulated synapses.^28^ KO of either or both isoforms decreased the number of AR events without affecting the kinetics of synchronous EPSCs (Figures 2D–2F). As with climbing fibers, the reduction in AR could not be accounted for by changes in quantal size because there was no difference in amplitudes or kinetics of spontaneous EPSCs recorded from MLIs, which receive glutamatergic input only from parallel fibers (Figures S1D–S1F). Compared to WTs, the number of AR events per stimulus decreased by 23% in *Syt3* KOs, and 49% in *Syt7* KOs (Figures 2E and 2F). In DKO synapses, AR was reduced by 71%, indicating that together, SYT3 and SYT7 account for the majority of AR from parallel fibers.

Although SYT3 and SYT7 share high affinities for Ca^2+^, they unbind from Ca^2+^ with vastly different kinetics,^29^ suggesting the two isoforms could promote AR on different timescales. Consistent with this scenario, we found that AR decayed more slowly in *Syt3* KOs and more rapidly in *Syt7* KOs (Figure 2F). The prolonged time course of AR in *Syt3* KOs could reflect SYT7 remaining bound to Ca^2+^ for longer after each stimulus, and vice versa in *Syt7* KOs. Surprisingly, AR decay was slowed in DKOs relative to WTs, suggesting that the remaining AR in DKO neurons is driven by a sensor that unbinds from Ca^2+^ slowly or possesses an unusually high affinity.

MLIs are small cells with high input resistances; thus, even a single quantal event can modulate the firing of these neurons.^30^ To test whether AR driven by SYT3 and SYT7 affects MLI firing, we performed current-clamp recordings from MLIs while stimulating parallel fibers. For these experiments, MLIs were first voltage clamped, and stimulation intensity was adjusted to elicit 700–800 pA EPSCs. MLIs were then current clamped to −70 mV, and we measured the reliability of parallel fiber stimulation in inducing postsynaptic action potentials (Figure 2G). In all genotypes, parallel fiber stimulation elicited short-latency action potentials in 100% of trials. In WTs, stimulations drove a second delayed action potential in 44% of trials. In Syt3 and Syt7 KOs, the probability of a second action potential was reduced by 28% and 41% relative to WT, respectively (Figure 2H). DKOs showed drastically reduced delayed firing probability by 73% relative to WT. These results provide additional evidence that AR driven by SYT3 and SYT7 can alter the number and timing of postsynaptic action potentials.

To investigate the Ca^2+^ dependence of AR that persists in DKO synapses, we bath applied 100 μM EGTA-AM, a membrane-permeant Ca^2+^ chelator that reduces the amplitude and duration of presynaptic residual Ca^2+^ signals.^3^ In DKO synapses, EGTA-AM further reduced AR by ~60% (Figure S5). The ability of EGTA-AM to block AR in DKOs suggests that the remaining AR is triggered by residual Ca^2+^ and driven by a sensor with high Ca^2+^ affinity. Alternatively, AR in DKOs could result from the release of vesicles positioned further from Ca^2+^ channels,^31^ as EGTA constrains the spatial extent of local Ca^2+^ signals.

### SYT3 and SYT7 are required for AR onto hippocampal interneurons

**Figure 3.**
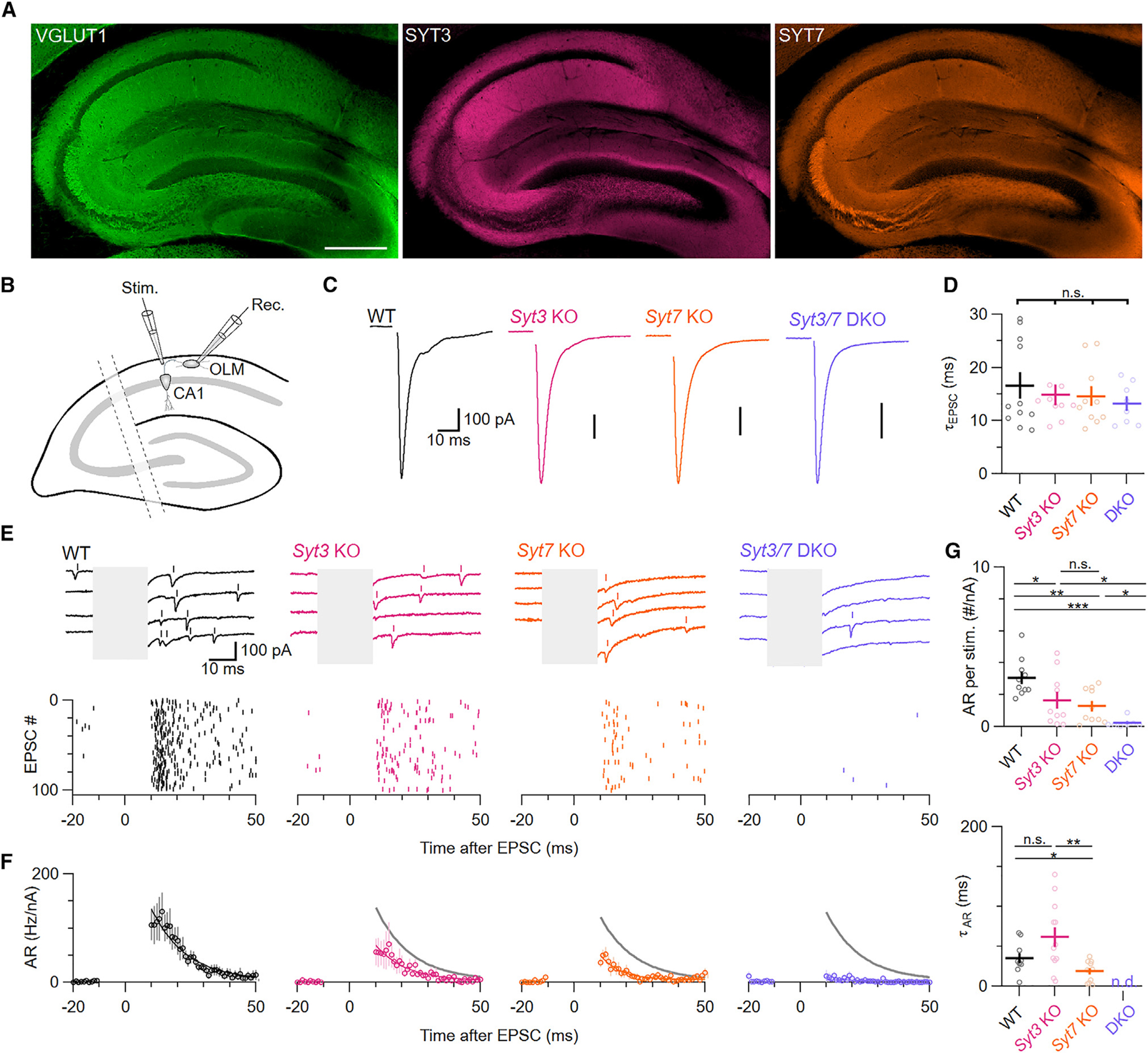
SYT3 and −7 are required for AR onto O-LM interneurons (A) Immunolabeling for VGLUT1 (green), SYT3 (magenta), and SYT7 (orange) in the hippocampus. Scale bar: 500 μm. (B) Schematic for recording EPSCs from CA1 pyramidal neurons (CA1) onto stratum oriens-lacunosum-moleculare (O-LM) interneurons. (C) Representative average of 100 EPSCs elicited by stimulating afferent CA1 axons. Stimulus artifacts have been blanked for clarity. (D) Decay time constants of synchronous EPSCs (*p* = 0.71). (E) Examples of individual synaptic responses (top). Detected AR is indicated as vertical bars above each raw trace. Raster plots of detected AR events for 100 consecutive trials (bottom). (F) Time course of AR normalized to the number of activated inputs (see the STAR Methods). Gray lines depict exponential fit to WT data for comparison. (G) Normalized number of AR events per stimulus (top; WT vs. *Syt3* KO: *p* = 0.011; WT vs. *Syt7* KO: *p* = 0.002; WT vs. DKO: *p* = 6.93 × 10^−6^; *Syt3* KO vs. *Syt7* KO: *p* = 0.70; *Syt3* KO vs. DKO: *p* = 0.01; *Syt7* KO vs. DKO: *p* = 0.01; critical α: 0.0167) and decay time constant of AR (bottom; WT vs. *Syt3* KO: *p* = 0.04; WT vs. *Syt7* KO: *p* = 0.015; *Syt3* KO vs. *Syt7* KO: *p* = 0.004; critical α: 0.025). Averages shown with error bars represent mean ± SEM. Normal distribution was verified with Shapiro-Wilk tests. Subsequently, statistical significances were evaluated using ANOVA, followed by two-tailed Student’s t tests and a post hoc Šidák correction for multiple comparisons where necessary. The number of experiments is shown in Table S1.

To test whether SYT3 and SYT7 promote AR in other brain regions, we performed recordings in the CA1 region of hippocampus, another area where both proteins are expressed in abundance (Figure 3A). We visually identified putative oriens-lacunosum-moleculare (O-LM) interneurons by their shape and location near the border of stratum oriens and alveus. O-LM cells receive highly facilitating inputs from CA1 pyramidal cells, which express both *Syt3* and *Syt7*. To record AR, putative interneurons were voltage clamped and CA1 axons were stimulated in the adjacent stratum oriens.^32^ Similar to parallel fiber synapses, KO of either isoform decreased the number of AR events (Figures 3C–3F) without affecting the kinetics of synchronous EPSCs or the amplitude or frequency of spontaneous EPSCs (sEPSCs) (Figures S1G–S1I). Compared to WT synapses, *Syt3* and *Syt7* KOs exhibited 49% and 61% reduced AR, respectively (Figures 3E–3G). Remarkably, AR was virtually absent in DKO synapses (92% decrease relative to WT). We again noted a trend in the rate at which AR decayed after each stimulus, with AR decaying the slowest in *Syt3* KOs and the fastest in *Syt7* KOs. These results show that SYT3 and SYT7 act in concert to promote AR in multiple brain regions.

### *Syt3/Syt7* DKO mice show no compensatory changes

Previous studies reported that constitutive *Syt7* KO mice exhibit normal synaptic Ca^2+^ signaling, baseline release properties, and gene expression.^11,15,16,28,33^ However, similar analyses have not been performed on *Syt3* KO or DKO animals. Given that SYT3 and SYT7 are the most abundant high-affinity SYTs in the brain^34^ and serve overlapping synaptic functions,^21^ deleting both isoforms might lead to compensatory changes in neuronal function or gene expression. Thus, we evaluated *Syt3* KO and DKO animals for compensatory changes that could indirectly affect AR.

**Figure 4.**
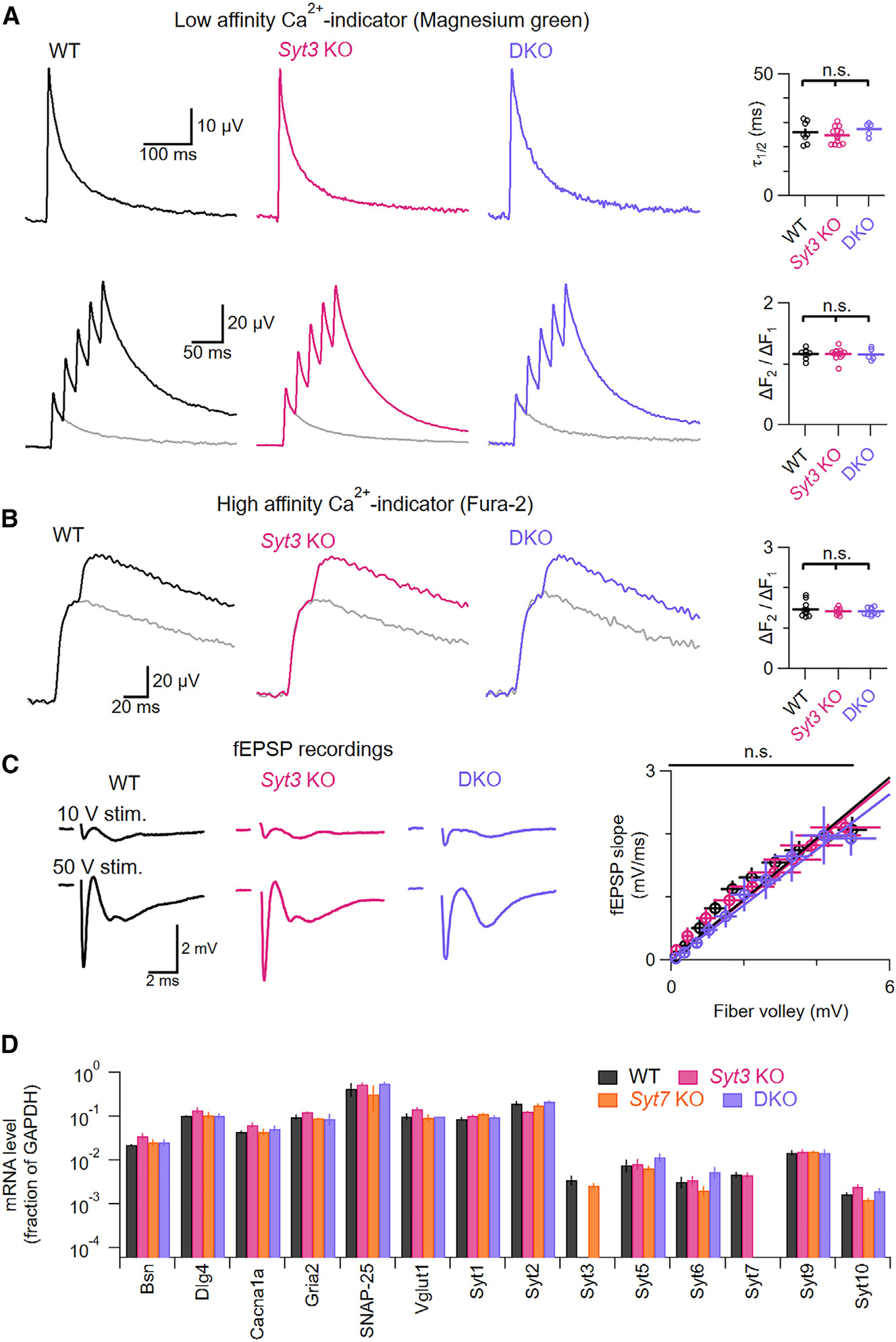
Knockout of *Syt3* and/or *Syt7* does not alter presynaptic Ca^2+^ signals, initial release probability, or synaptic gene expression (A) Presynaptic Ca^2+^ signals evoked by a single stimulus (top) and a train of five stimuli at 50 Hz superimposed over a single stimulus (bottom) recorded from parallel fibers loaded with a low-affinity Ca^2+^ indicator, Magnesium Green (left). Half-decay time constants after a single stimulus (top; *p* = 0.39) and amplitude ratio (bottom; *p* = 0.95) of the first two Ca^2+^ signals in a train (right). (B) Ca^2+^ signals evoked by a single stimulus (gray) or two stimuli at 50 Hz recorded from parallel fibers loaded with a high-affinity Ca^2+^ indicator, Fura-2 (left). Ratio of the fluorescence increase to the two stimuli (right; *p* = 0.54). (C) Representative fiber volleys and fEPSPs recorded extracellularly in the molecular layer during parallel fiber stimulation at different intensities. Average ratio of fEPSP slopes to fiber volley amplitude for stimulation intensities from 10 to 100 V (right; *Syt3* KO: *p* = 0.79; DKO: *p* = 0.87). (D) mRNA levels (normalized to GADPH levels) of Ca^2+^-binding synaptotagmin isoforms and other genes involved in synaptic transmission. Measurements are from cerebellar tissue from 4 mice per genotype with 3 technical replicates per animal. mRNA quantification for additional genes is shown in Figure S6. Averages shown with error bars represent mean ± SEM. Normal distribution was verified with Shapiro-Wilk tests. Subsequently, statistical significances were evaluated using ANOVA (A and B) or Kolmogorov-Smirnov tests (C). The number of experiments is shown in Table S1. See Table S2 for primers.

Because AR is highly dependent on presynaptic residual Ca^2+^, we first measured the time course of residual Ca^2+^ by loading parallel fibers with a low-affinity fluorescent indicator (Magnesium Green). Across genotypes, residual Ca^2+^ decayed with similar kinetics after single stimuli, while trains of stimuli evoked the same increase in Ca^2+^ during repeated stimulation. Thus, genotypic differences in AR do not result from altered residual Ca^2+^ kinetics or use dependence (Figure 4A). We performed similar experiments using fura-2, a high-affinity indicator that partially saturates in response to action-potential-induced Ca^2+^ influx. Because the degree of fura-2 saturation is sensitive to total presynaptic Ca^2+^ influx, it is possible to detect changes in Ca^2+^ influx by measuring the ratio of fluorescence increases to the first and second stimuli.^35^ Fura-2 response ratios were similar across genotypes, suggesting similar amounts of presynaptic Ca^2+^ influx (Figure 4B).

We next assessed KO synapses for changes in initial release probability, which could indirectly affect the balance between synchronous and AR. We recorded field excitatory postsynaptic potentials (fEPSPs) and presynaptic fiber volleys produced by stimulating parallel fiber axons. Fiber volleys scale with the number of activated axons, while fEPSPs report the total synaptic efficacy of all activated axons. Hence, the ratio of fEPSP to fiber volley is proportional to the release per activated axon. This ratio was similar across genotypes, suggesting no differences in initial release probability (Figure 4C). Finally, we determined whether deleting *Syt3* and/or *Syt7* alters gene expression by performing RT-qPCR in cerebellar tissue for critical genes involved in synaptic transmission (P/Q-type Ca^2+^ channels, soluble *N*-ethylmaleimide-sensitive factor attachment protein receptor [SNARE] proteins, ionotropic glutamate receptors) or other Ca^2+^-binding SYT isoforms (Figures 4D and S6). We did not detect major changes in gene expression from any genotype. In summary, *Syt3* KO and DKO animals appear to lack major compensatory changes that might indirectly alter AR, which suggests that SYT3 and SYT7 promote AR directly.

### SYT3 and SYT7 are potent sensors for Sr^2+^-induced AR

AR is often studied by replacing extracellular Ca^2+^ with Sr^2+^, which dramatically reduces synchronous release while increasing the magnitude and duration of AR.^36^ Two competing theories have been advanced to explain why Sr^2+^ enhances AR: in one theory, Sr^2+^ activates the high-affinity sensor more effectively than the sensor for synchronous release.^5^ A second theory posits that Sr^2+^ is equally ineffective at activating both sensors, but intracellular Ca^2+^ buffers are unable to capture free Sr^2+^, leading to larger “residual Sr^2+^” signals that promote more AR.^37^ Notably, SYT3 may bind Sr^2+^ with higher affinity than other SYTs^38^ and might thus be uniquely suited to promote Sr^2+^-induced AR.

**Figure 5.**
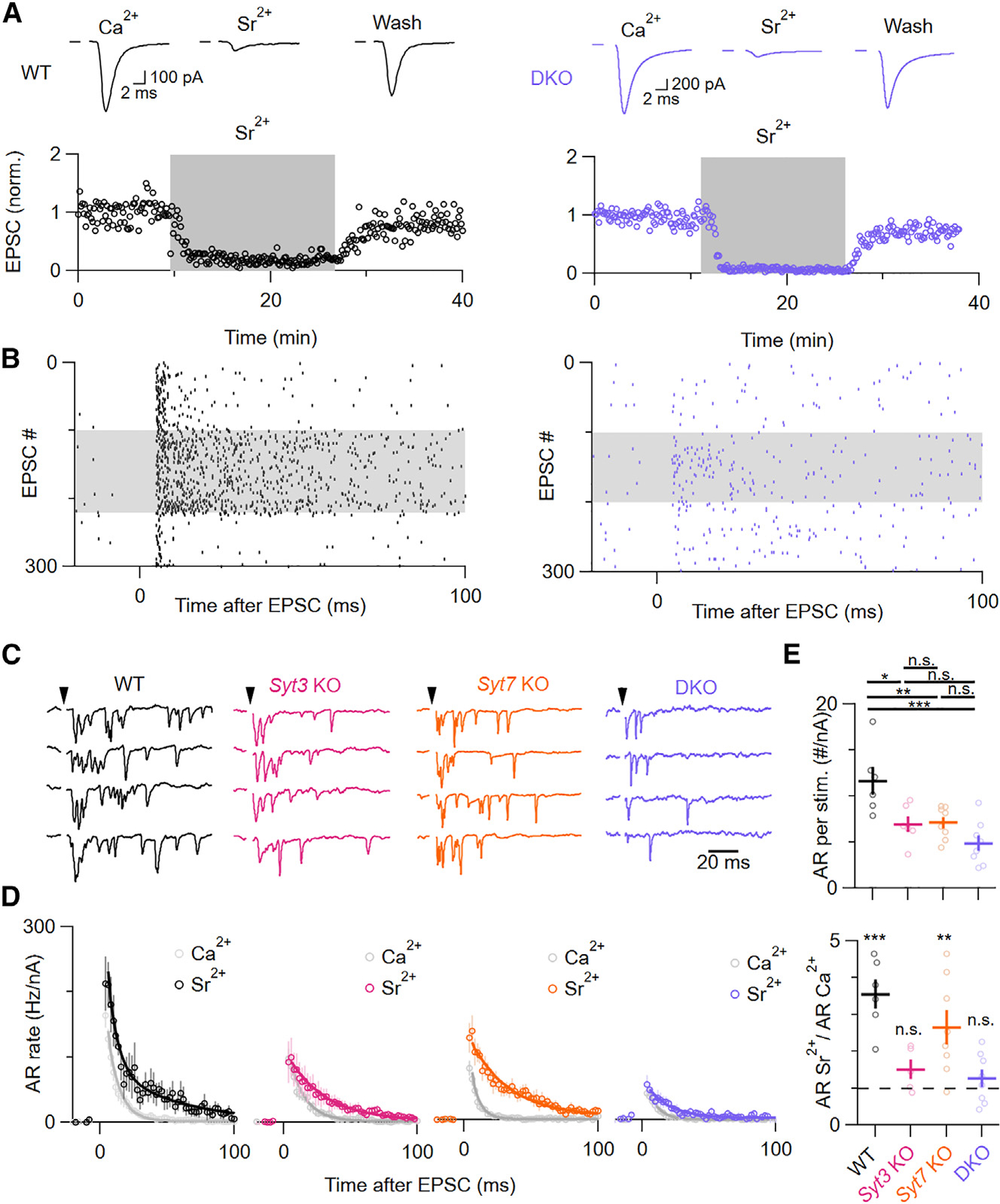
Sr^2+^-induced AR is predominantly driven by SYT3 (A) Representative average of 100 EPSCs with stimulation artifacts blanked in MLIs elicited by parallel fiber stimulation at 0.125 Hz in WT (black) and DKO (purple) animals in Ca^2+^, Sr^2+^, and after Sr^2+^ wash-out (top). EPSC amplitudes from a representative recording normalized to the first 100 EPSCs in Ca^2+^ (bottom). (B) Raster plot showing AR events during 300 consecutive trials from the recordings shown in (A). (C) Example traces showing AR in Sr^2+^ after stimulation, with stimulation artifacts blanked and indicated as black arrowheads. (D) Average time course of AR in Sr^2+^ for WT (black), SYT3 KO (magenta), SYT7 KO (orange), and SYT3/7 DKO (purple) synapses superimposed with the respective AR in Ca^2+^ (Figure 2G, gray) normalized to the number of activated parallel fibers in Ca^2+^ (see STAR Methods). Averages were fit using double exponentials in WT in Sr^2+^ and single exponentials for all other conditions. (E) Normalized number of AR events per stimulus (WT vs. *Syt3* KO: *p* = 0.01; WT vs. *Syt7* KO: *p* = 0.0009; WT vs. DKO: *p* = 1.21 ×3 10^−4^; *Syt3* KO vs. *Syt7* KO: *p* = 0.88; *Syt3* KO vs. DKO: *p* = 0.11; *Syt7* KO vs. DKO: *p* = 0.05; critical α: 0.0167) and ratio of AR in Sr^2+^ and Ca^2+^ (WT: *p* = 0.019; SYT3 KO: *p* = 0.18; SYT7 KO: *p* = 0.026; DKO: *p* = 0.36). Normal distribution was verified with Shapiro-Wilk tests. Subsequently, statistical significances were evaluated using ANOVA, followed by non-paired (AR per stim) and paired (ratio of AR per stimulus in Sr^2+^ and Ca^2+^) two-tailed Student’s t tests and a post hoc Šidák correction for multiple comparisons where necessary. The number of experiments is shown in Table S1.

We tested this possibility at the parallel fiber-to-MLI synapse, where Sr^2+^-induced AR has been described in detail.^28^ During recordings, normal bath solution was exchanged for artificial cerebrospinal fluid containing 4 mM Sr^2+^ and 2 mM EGTA (Figure 5A). Because EGTA has a higher affinity for Ca^2+^ than Sr^2+^, this solution is expected to contain 2 mM free Sr^2+^ and be nominally free of extracellular Ca^2+^. All mutant genotypes exhibited significantly less AR in the presence of Sr^2+^ compared to WTs (Figures 5C–5E). However, in WT and *Syt7* KO synapses, Sr^2+^ still produced a ~2-fold increase in total AR. Surprisingly, Sr^2+^ did not significantly increase AR from *Syt3* KOs or DKOs, suggesting that SYT3 is required for Sr^2+^-induced AR enhancement in parallel fibers.

We performed similar experiments at climbing fiber synapses, where Sr^2+^ also increases AR.^18^ *Syt3* KO climbing fibers showed 79% fewer AR events than WTs, which led to a faster decay of the averaged EPSC waveform (Figure S7). Sr^2+^ wash-in also caused a stronger reduction of synchronous EPSC amplitudes in *Syt3* KOs, suggesting that SYT3 binds to Sr^2+^ rapidly enough to contribute to synchronous release. Taken together, these results suggest that SYT3 and SYT7 both contribute to AR in the presence of Sr^2+^. However, SYT3 appears to play a larger role, consistent with its unusually high affinity for Sr^2+^.

## DISCUSSION

### Multiple Ca^2+^ sensors shape release kinetics

At least 8 Ca^2+^-binding SYT isoforms are expressed in the central nervous system,^39^ but whether they all participate in Ca^2+^-triggered release remains unknown. SYT1 clearly acts as the primary Ca^2+^ sensor for synchronous vesicle fusion in central synapses, particularly in the mammalian forebrain.^40^ However, the first recordings from murine SYT1 KO neurons showed AR to be unaffected, despite synchronous release being severely impaired. Geppert et al.^6^ advanced a two-sensor model, where SYT1 drives synchronous release, and the recently discovered SYT3 was proposed to mediate AR. Three decades later, our results finally confirm their prescient hypothesis.

Two-sensor models have been successful in explaining the kinetics and Ca^2+^ dependence of neurotransmitter release.^37,41–43^ Our results suggest that these models must be extended to incorporate more sensors (SYT1, −3, and −7 at parallel fibers and synapses onto hippocampal interneurons). SYT3 and SYT7 bind to Ca^2+^ with different affinities and kinetics, and the relative abundance of each isoform could tune release kinetics at different synapse types. SYT3 unbinds from Ca^2+^ rapidly, while SYT7 unbinds extremely slowly.^29^ The two isoforms might thus promote fast and slow forms of AR, respectively. Indeed, we found that the duration of AR was prolonged in MLIs in *Syt3* KOs and accelerated in *Syt7* KOs in both MLIs and hippocampal interneurons. Our finding that synaptically induced action potentials were less frequent and delayed in KOs supports the notion that AR affects the timing of postsynaptic firing and thereby influences information transfer within circuits.

Notably, we found that DKO parallel fibers retained a small component of AR, which decayed slowly and was reduced by EGTA-AM. This suggests that AR involves other, unknown high-affinity Ca^2+^ sensors. DOC2 is perhaps the best-studied and strongest candidate to serve as an additional sensor, as knockdown/KO of *Doc2* reduces AR from cultured hippocampal synapses.^44,45^ DOC2 proteins bind Ca^2+^ with high affinity and are structurally similar to SYTs. DOC2a is proposed to be particularly effective in mediating AR after a single stimulus, whereas some studies report that SYT7 promotes AR only after stimulus trains.^11,13,17^ Wu and colleagues^12^ found that *Doc2α* KO synapses showed reduced AR after single stimuli, but AR deficits appeared in *Syt7* KO synapses only during prolonged 10–20 Hz stimulus trains. However, it remains unclear whether SYT7 affects AR variably at different synapses or whether subtle changes in AR are simply difficult to resolve. Stimulus trains may be required in some preparations to raise AR to detectable thresholds. Here, we find that SYT3 and SYT7 promote robust AR after single stimuli, as other studies have shown.^16,28^

### Mechanism of SYT-triggered AR

It remains unclear as to how SYT3/7 promote the delayed release of vesicles. By combining ultrastructure with physiological recordings, Wu and colleagues^12^ showed that SYT7 transiently docks vesicles following an action potential and proposed that DOC2α subsequently drives the AR of these vesicles. Similarly, we previously used modeling to infer that SYT3 transiently docks vesicles to sustain synchronous release.^21^ In these models, SYT3/7 pull vesicles closer to the active zone membrane for the duration of the residual Ca^2+^ signal. This prepares vesicles for release but does not trigger fusion directly. Such transient docking is supported by biophysical models of short-term plasticity, and ultrastructure studies that show fewer docked vesicles in *Syt7* KO synapses for tens of milliseconds after action potentials.^46^ Transient docking could explain how SYT3 and SYT7 enhance asynchronous and synchronous release during short-term plasticity: by briefly increasing the pool of releasable vesicles that are driven to fusion by DOC2 or SYT1.

Alternatively, SYT1, −3, and −7 could all act similarly to trigger vesicle fusion.^47^ This scenario suggests that multiple SYT isoforms interact with the release machinery of each vesicle. Several lines of evidence support this possibility: SYT1, −3, and −7 have all been shown to bind to SNARE proteins. A SYT1-SNARE-complexin crystal structure suggested that two distinct interfaces can bind SYTs. The “primary” interface only accommodates the “fast” isoforms SYT1, −2, and −9. A “tripartite” interface could accommodate any SYT or DOC2 protein.^48^ *In vitro* experiments suggest that as many as 12 SNARE complexes assemble beneath a single docked vesicle.^49^ Taken together, these results paint a picture where many SYTs of any isoform could assemble with the macromolecular release machinery complex. Multiple copies of SYT1 interacting with SNARE complexes^50^ can explain the cooperativity of Ca^2+^-dependent fusion,^51^ and the models where multiple copies of SYT1 and SYT7 act cooperatively to lower the fusion barrier have been able to recapitulate both short-term plasticity and AR.^52^ As evidence increasingly suggests important roles for SYT3 and SYT7 in release, it is apparent that more work is needed to define the interaction partners that support their function.

### Limitations of the study

Our study has several limitations: first, although the reduction in AR we observed after genetically depleting *Syt3* and/or *Syt7* does not appear to be caused by changes in baseline synaptic properties or neuronal excitability, our use of constitutive KO animals raises the possibility for off-target genetic or developmental compensation. In the future, the conclusion that SYT3 and SYT7 contribute directly to AR would be strengthened by genetic approaches that reduce the possibility of compensatory effects. This could involve targeted depletion of SYTs from presynaptic cells or rescue expression in adult KO animals. Second, our experiments do not define the extent to which SYT3 and SYT7 promote AR *in vivo*. Most recordings were performed at room temperature in mildly elevated extracellular Ca^2+^ (2 mM). We chose these conditions to mirror previous studies and improve our ability to detect AR. Although AR has historically been studied by recording under these conditions, recordings performed in more physiological conditions could more accurately determine how AR affects synaptic information transfer *in vivo*. Finally, we confined our experiments to well-characterized glutamatergic synapses. More work is needed to discover whether SYT3 and SYT7 promote the AR of GABA and other neurotransmitters.

## STAR★METHODS

### RESOURCE AVAILABILITY

#### Lead contact

Requests for information, resources, and reagents should be directed to the lead contact, Skyler L. Jackman (jackmans@ohsu.edu).

#### Materials availability

Transgenic mouse lines used in this study are either publicly available or will be shared within the limits of existing material transfer agreements.

#### Data and code availability

- Data are available upon request from lead contact.
- Custom code used for analysis of asynchronous release in this study is available on GitHub (https://github.com/skylerjackman).
- Any additional information required to reanalyze the data reported in this work is available from the lead contact upon request.

### EXPERIMENTAL MODEL AND STUDY PARTICIPANT DETAILS

#### Sample sizes

Sample sizes were selected to match or exceed typical sample sizes reported in similar studies. Power analysis was not used to determine sample sizes in advance. All electrophysiology and subsequent analyses were performed blind to genotype. No other blinding was performed.

#### Animals

All mice were handled in accordance with NIH guidelines and protocols approved by Oregon Health & Science University’s Institutional Animal Care and Use Committee. *Syt3* KO,^53^ *Syt7* KO,^54^ *Syt*3/7 double KO (DKO), and WT animals of either sex from homozygous or heterozygous breeding pairs (littermates) were used. DKOs were generated from heterozygous breeding pairs.

### METHOD DETAILS

#### Acute slice preparation

Acute 270 μm-thick brain slices were prepared from P30–80 animals. Animals were anesthetized with isoflurane and euthanized. Brains were quickly removed into ice-cold cutting solution containing (in mM): 125 Choline-Cl, 25 NaHCO_3_, 10 glucose, 2.5 KCl, 1.25 NaH_2_PO_4_, 2 Na-pyruvate, 3 (3)-myo-inositol, 4.4 ascorbic acid, 7 MgCl_2_, 0.5 CaCl_2_, bubbled continuously with 95% O_2_/5% CO_2_. Slices were prepared using a Leica VT1200S vibratome and transferred to a holding chamber with ACSF containing (in mM): 125 NaCl, 25 NaHCO_3_, 10 glucose, 2.5 KCl, 1.25 NaH_2_PO_4_, 2 Na-pyruvate, 3 (3)-myo-inositol, 4.4 ascorbic acid, 1 MgCl_2_, 2 CaCl_2_, bubbled continuously with 95% O_2_/5% CO_2_. Slices were allowed to recover at 35°C for 10 min, then stored at room temperature (24 ± 1°C) prior to recording. MLI and Purkinje cell recordings were performed in the vermis of transverse and sagittal cerebellar slices, respectively. Hippocampal interneuron recordings were performed in transverse hippocampal slices in the CA1 region of stratum oriens. A cut was made between CA3 and CA1 to prevent recurrent activity from CA3 axons in the presence of picrotoxin.

#### Electrophysiology

Recordings were performed at room temperature unless otherwise stated. Slices were continuously perfused with oxygenated ACSF at ~3 mL/min. To isolate AMPAR-driven responses, 100 μM of Picrotoxin was added to the ACSF. Whole-cell recordings were obtained using a EPC10/2 patch-clamp amplifier (HEKA Elektronik) or an Multiclamp 700B (Axon Instruments). For MLI recordings, putative stellate cells were patched in the outer third of the molecular layer with borosilicate glass electrodes (2–3 MΩ). Cells were voltage-clamped at −70 mV, ACSF-filled monopolar glass stimulation electrodes (2–3 MΩ) were placed in the molecular layer 50–100 μm from the targeted cell, and brief (100μs) square voltage pulses of were used to stimulate single action potentials in presynaptic axons. This short stimulation was chosen to be within the refractory period of voltage gated sodium channels to ensure only a single action potential was induced in stimulated axons. For hippocampal recordings, putative O-LM interneurons were targeted due to their location at the border of the oriens/alveus, and oval shape and orientation,^55^ and patched with borosilicate glass electrodes (3–4 MΩ). Cells were voltage-clamped at −70 mV, ACSF-filled monopolar glass stimulation electrodes (2–3 MΩ) were placed 50–100 μm lateral to the targeted cell,^32^ and brief square voltage pulses of 100μs length were used to stimulate single action potentials in presynaptic axons. For Purkinje cell recordings, Purkinje cells were patched with 1.2–2 MU pipettes, and ACSF-filled monopolar glass stimulation electrodes (2–3 MΩ) were placed in the granule cell layer 50–100 μm from the targeted cell. To ensure no contamination from parallel fiber inputs, stimulation intensity was gradually increased to produce an all-or-none climbing fiber response. The recording pipette contained (in mM): 130 Cs-gluconate, 10 CsCl, 5 EGTA, 10 HEPES, 10 TEA-Cl, 5 Na-phosphocreatine, 4 Mg-ATP, 0.5 Na-GTP, 5 QX-314 (300 mOsm, pH adjusted to 7.28 with CsOH). For on-cell recordings, recording pipettes contained ACSF. Current-clamp recordings were performed with a pipette solution containing (in mM): 140 K-gluconate, 10 HEPES, 5 EGTA, 2 KCl, 10 ATP-Mg, 5 GTP-Na2, 2 Na-phosphocreatine (305 mOsm, pH adjusted to 7.25 with KOH). As we found that pacemaking activity in Purkinje cells was impaired at room temperature, we elevated the temperature to 34 ± 1°C for current clamp experiments testing Purkinje cell excitability. Field recordings were performed in sagittal cerebellar slices with ACSF-filled pipettes being placed in the molecular layer ~20 μm from the Purkinje cell layer and 100–200 μm deep within a slice. Stimulation electrodes then were placed on the surface right above the recording pipette. In all experiments, data were acquired with a sampling rate of 20 or 50 kHz and low-pass Bessel-filtered at 2.9 kHz. In Purkinje cell recordings, series resistance was compensated by at least 80%. Recordings were excluded if series resistance was >10 MΩ prior to compensation, or if series resistance changed by > 20% during the experiment. EPSC amplitudes were calculated from the baseline 1 ms before a stimulation artifact. To isolate AR, baseline spontaneous release was determined 500 ms before and 1–5 s after stimulation of inputs and subtracted. Data in figures represent mean ± SEM. Inter-stimulus-intervals to evoke asynchronous release were 8 s for all synapses. Analysis of EPSC amplitudes and exponential fits of data were performed in IgorPro 9 (WaveMetrics).

#### AR and spontaneous EPSC detection

For AR detection, the decaying phase of EPSCs from 90% of the peak was fit using a double-exponential and subtracted from the baseline in IgorPro 9. Recordings with AR and spontaneous EPSCs were low-pass filtered at 0.8 kHz, and the first derivative was generated to improve signal-to-noise ratio for detecting quantal events.^56^ For MLI and O-LM recordings, the detection threshold was set to 2 × RMS (RMS detected 1–5 s after the stimulation artifact) or 20 pA/ms, whichever was greater. Peaks of quantal events were detected up to 1 ms after the local minima of differentiated traces. Events with amplitudes <20 pA and within 1 ms of another event were excluded from analysis. For Purkinje cell recordings, thresholds were set to 35 pA/ms and 35 pA. Because of the difficulty of detecting AR in the decaying phase of EPSCs, 5 ms, 10 ms and 20 ms after the peak of average EPSCs were excluded from analysis in MLIs, O-LMs, and climbing fibers, respectively. Code for this analysis is available on GitHub (https://github.com/skylerjackman).

#### Ca^2+^-imaging

To measure presynaptic residual Ca^2+^ transients,^3^ glass pipettes were filled with ACSF, and either Magnesium Green AM or Fura-2 a.m. (240 μM, Invitrogen). 1% fast green was added for visualization during loading. Pipettes were placed into the molecular layer of transverse cerebellar slices, positive pressure was applied, and a vacuum pipette was positioned downstream to remove excess indicator. Both pipettes were withdrawn after brief loading (~3 min), and slices were incubated at room temperature for at least 1 h to allow indicator diffusion along parallel fiber axons. Imaging was performed >500 μm from the injection site using a 60× objective and custom-built photodiode. A bipolar stimulus electrode was placed ~300 μm from the imaging site, and Magnesium Green and Fura-2 were excited using a 488 nm or 405 nm LED, respectively (Thorlabs). All imaging was performed at room temperature.

#### Immunohistochemistry

Mice were anesthetized with ketamine/xylazine (100/10 mg/kg) and transcardially perfused with 4% paraformaldehyde (PFA) in PBS. Brains were removed and post-fixed overnight at 4°C. 20–50 mm slices were prepared using a Leica VT1000S vibratome, permeabilized for 1 h in vehicle (PBS, 10% normal goat serum, 0.3% Triton X-100), then incubated overnight at 4°C in vehicle with primary antibodies (rabbit anti-Syt3, 1:500, Synaptic Systems 105133; guinea pig anti-vGlut1, 1:500, Synaptic Systems 135304; guinea pig anti-vGlut2, 1:500, Synaptic Systems 135404; mouse anti-bassoon, 1:500, ThermoFisher, SAP7F407; mouse anti-PSD95, 1:500, Antibodies Inc., 75–028). After 4 washes for 5 min in vehicle, slices were incubated with secondary antibodies in vehicle for 2 h at room temperature (goat-*anti*-rabbit Alexa 546, 1:500, ThermoFisher A-11035; goat-*anti*-rabbit Cy3, 1:500, Jackson, 111–165-003; goat-*anti*-guinea pig CF633, 1:400, Biotum 20129; goat-*anti*-mouse AF488, 1:500, Jackson, 115–545-003). After 4 washes in PBS, slices were mounted in Prolong Gold. Fluorescent images were acquired using a Zeiss LSM980 (Airyscan) or Zeiss Elyra7 (SIM) equipped with a 63× objective.

#### RT-PCR

Following transcardial perfusion, cerebella from adult mice brain were dissected. Total RNA was isolated using a Purelink RNA mini kit (ThermoFisher) according to manufacturer’s instructions and quantified using Nanodrop One Spectrophotometer (Thermo Scientific). First strand cDNA was reverse transcribed from 2μg RNA using a High-capacity cDNA Reverse Transcription kit (Applied Biosystems) with and without reverse transcriptase to compute for negative control. Quantitative RT-PCR was performed using PowerUp SYBR Green (Applied Biosystems) in QuantStudio 7 Flex real-Time PCR instrument (Applied Biosystems). Primers were designed from ThermoFisher, and the relative expression of each gene was normalized to GAPDH. Data were analyzed using 2-ΔCt method.

#### snRNA-seq library analysis

For snRNA-seq analyses, published single nuclei datasets from cerebellar granule cells, CA1 pyramidal neurons and Purkinje cells^26^ were sorted with respect to the number of total detected transcripts per nucleus. Data were binned (20000 transcripts per bin for granule cells, 10000 transcripts per bin for CA1 neurons and Purkinje cells) and the fraction of nuclei positive for *Syt3* and/or *Syt7* was calculated per bin.

### QUANTIFICATION AND STATISTICAL ANALYSIS

#### Statistical analysis

Statistical analyses were performed using Microsoft Excel or IgorPro 9. Normal distribution of samples was assessed using Shapiro-Wilk tests. If not otherwise stated, statistical significance for normally distributed data was tested using two-tailed Student’s t-tests for comparisons of 2 groups, or one-way ANOVA followed by two-tailed Student’s t-tests for comparisons of multiple groups. Cumulative distributions were compared using Kolmogorov-Smirnov tests. Critical significance thresholds were post-hoc Šidák corrected to account for multiple comparisons.

## Supplementary Material

1

## Figures and Tables

**KEY RESOURCES TABLE:** 

REAGENT or RESOURCE	SOURCE	IDENTIFIER

Antibodies		
Rabbit anti-SYT3	Synaptic Systems	Cat#:105133; RRID:AB_2617067
Guinea pig anti-VGLUT1	Synaptic Systems	Cat#:135304; RRID:AB_887878
Guinea pig anti-VGLUT2	Synaptic Systems	Cat#:135404; RRID:AB_887884
Mouse anti-SYT7	Neuromab	Cat#: 75265; RRID: AB_11030371
Goat anti-rabbit Alexa 546	ThermoFisher	Cat#: A-11035; RRID AB_2534093
Goat anti-guinea pig CF633	Biotium	Cat#:20129; RRID:AB_10852674
Goat anti-mouse AF488	ThermoFisher	Cat#: A-11001; RRID: AB_2534069
Chemicals, peptides, and recombinant proteins		
Picrotoxin	Indofine Chemical Company	Cat#: P-001
QX-314 chloride	HelloBio	Cat#: HB1030
Fura-2 a.m.	Invitrogen	Cat#: F1221
Magnesium Green AM	Invitrogen	Cat#: M3735
Deposited Data		
Custom code for for analysis of asynchronous release	GitHub:Skylerjackman/Mini-detection	https://doi.org/10.5281/zenodo.12600478
Experimental models: Organisms/strains		
C57BL/6J, Mus musculus	Jackson Laboratories	RRID: IMSR_JAX:000664
Syt7 Global Knockout B6.129S1-Syt7tm1Nan/J	Jackson Laboratories	RRID: IMSR_JAX:004950
Syt3 Global Knockout from B6; 129-Syt6tm1Sud Syt5tm1Sud Syt3tm1Sud Syt10tm1Sud/J	Jackson Laboratories	RRID: IMSR_JAX:008413
Oligonucleotides		
Custom Oligonucleotides for RT-PCR	This study	see Table S2
Software and algorithms		
Patchmaster v2x90.5	HEKA Elektronik	RRID: SCR_000034
MultiClamp 700B Commander	Axon Instruments	RRID: SCR_018455
IGOR Pro 9	WaveMetrics	RRID: SCR_000325
Microsoft Excel	Microsoft	RRID: SCR_016137
ImageJ/Fiji software	NIH/Fiji	RRID: SCR_002285

## Highlights

- Asynchronous neurotransmitter release is predominantly driven by SYT3 and SYT7
- SYT3 and SYT7 mediate asynchronous release on different timescales
- Asynchronous release prolongs the temporal window for postsynaptic firing
- SYT3 is a potent strontium sensor for asynchronous release
