# Supplementary material for: Synaptotagmins 3 and 7 mediate the majority of asynchronous release from synapses in the cerebellum and hippocampus
Source: Cell Rep. Author manuscript; Available in PMC 2024 Sep 18. (PMC11410144; doi:10.1016/j.celrep.2024.114595)
Supplement: 1 [file NIHMS2019539-supplement-1.pdf]

Cell Reports, Volume *43*

Supplemental information

Synaptotagmins 3 and 7 mediate the majority of asynchronous release from synapses in the cerebellum and hippocampus

Dennis J. Weingarten, Amita Shrestha, Daniel J. Orlin, Chloé L. Le Moing, Luke A. Borchardt, and Skyler L. Jackman

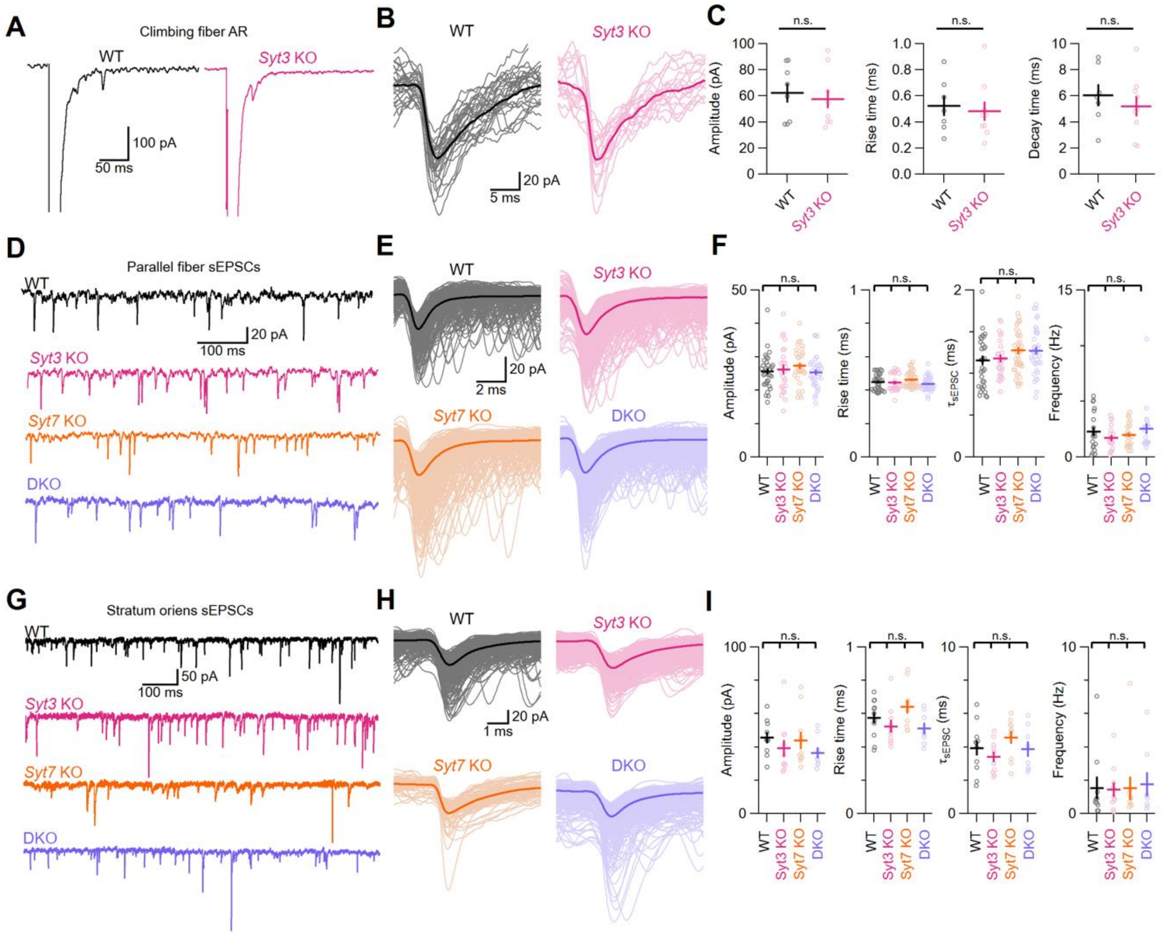

Figure S1 | Knockout of *Syt3* and/or *Syt7* does not alter quantal EPSC amplitudes or kinetics, related to Figures 1-3

A) Representative voltage-clamp recordings showing AR in Purkinje cells after climbing fiber stimulation in WT (black) and SYT3 KO (magenta) synapses. Only asynchronous events (10-200 ms after the EPSC) were used to determine quantal event kinetics because Purkinje cells also receive glutamatergic input from parallel fibers.

B) Averaged AR event waveforms.

C) Amplitude (P = 0.65), rise (20-80%; P = 0.25) and decay time constants (P = 0.40) of AR.

D) Representative voltage-clamp recordings in MLIs in the absence of stimulation in WT (black), *Syt3* KO (magenta), *Syt7* KO (orange), and *Syt3/7* DKO (purple) synapses.

E) Averaged spontaneous EPSC (sEPSC) waveforms in MLIs.

F) Amplitude (P = 0.50), rise (P = 0.12), decay time constants (P = 0.20) and spontaneous EPSC frequency (P = 0.39) of MLI sEPSCs.

G) Representative voltage-clamp recordings in O-LMs in the absence of stimulation in all genotypes.

H) Averaged spontaneous EPSC waveforms in O-LMs.

I) Amplitude (P = 0.48), rise (P = 0.08), decay time constants (P = 0.25), and spontaneous EPSC frequency (P = 0.98) sEPSCs in O-LMs.

Averages shown with error bars represent mean ± SEM. Normal distribution was verified with Shapiro-Wilk tests. Subsequently, statistical significances were evaluated using ANOVA. The number of experiments is shown in Table S1.

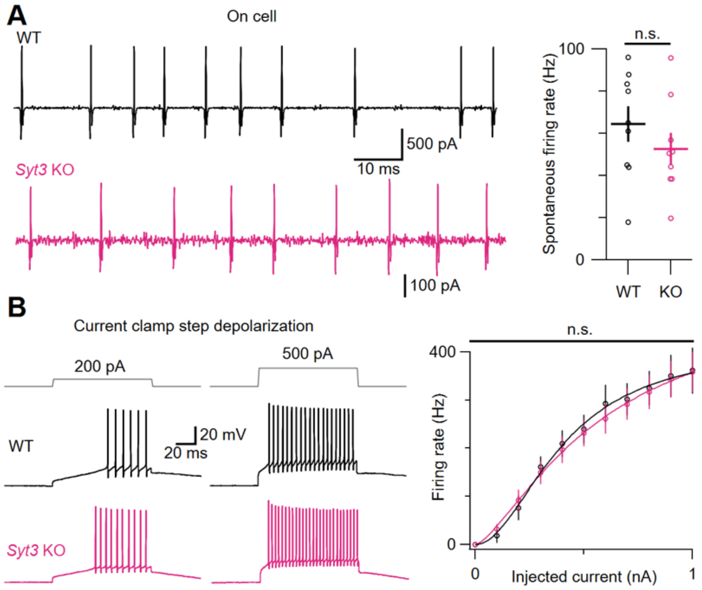

Figure S2 | SYT3 KOs Purkinje cells show similar spontaneous firing and excitability, related to Figure 1
A) Representative on-cell recordings of spontaneous firing in Purkinje cells in WT and SYT3 KO animals (left), and average spontaneous firing rate (right, $P = 0.31$).
B) Representative current-clamp recordings from Purkinje cells during 100 ms-long current injections from a holding potential of ~-80 mV (left) and average peak firing rates in Purkinje cells during current injections from 0.1 – 1 nA (right, $P = 0.87$). Data were fitted with sigmoid curves for visualization.
Averages shown with error bars represent mean ± SEM. Normal distribution was verified with Shapiro-Wilk tests. Subsequently, statistical significances were evaluated using two-tailed Student's t-tests in **A** and Kolmogorov–Smirnov tests in **B**. The number of experiments is shown in Table S1.

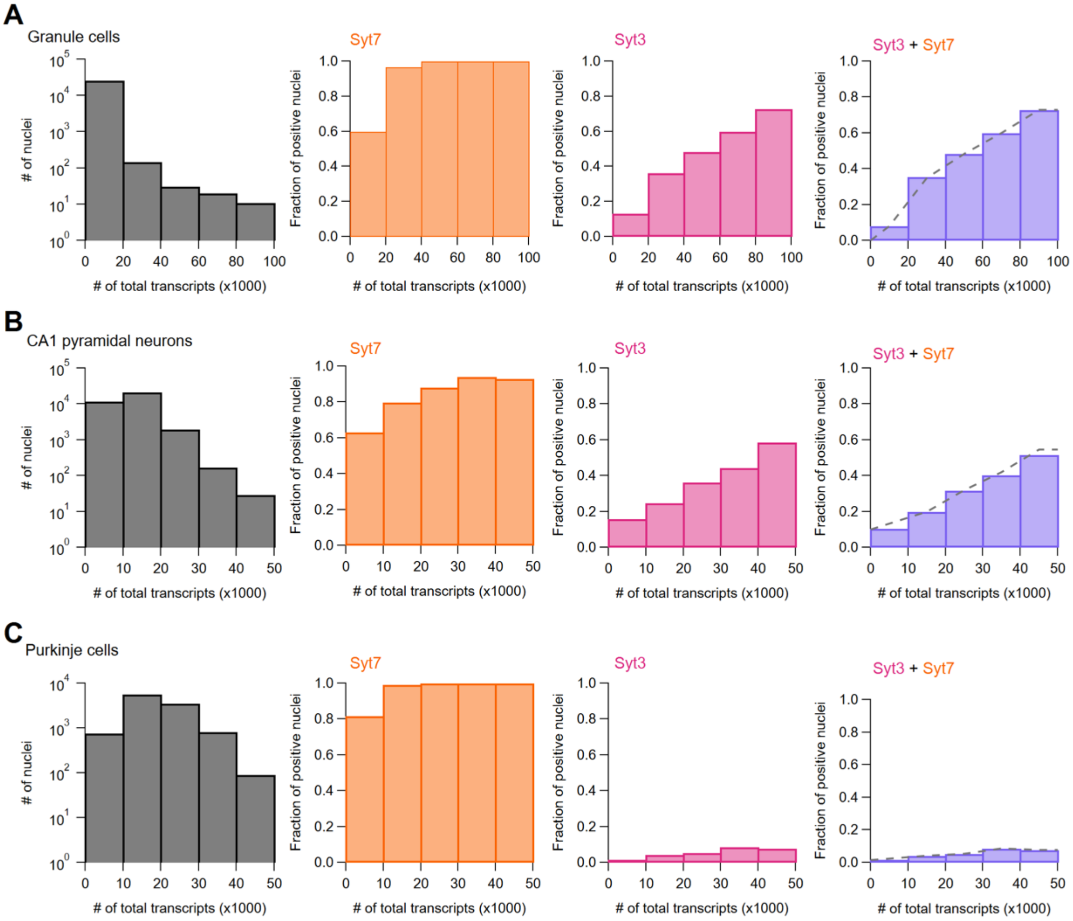

Figure S3 | *Syt3* and *Syt7* are co-expressed at the level of individual cerebellar granule cells and hippocampal CA1 neurons, but not in Purkinje cells, related to Figure 2 and 3
(A-B, left) Number of nuclei sorted by total number of transcripts detected using high throughput single-nuclei RNA-seq (Langlieb et al. 2023) for cerebellar granule cells (**A**), hippocampal CA1 pyramidal neurons (**B**) and Purkinje cells (**C**). (Right) Fraction of nuclei positive for Syt7 (orange), Syt3 (magenta) or both (purple). Dotted lines indicate expected probability of co-detecting *Syt3* and *Syt7* transcripts despite transcript dropout, calculated by multiplying the independent probability of detection of each gene.

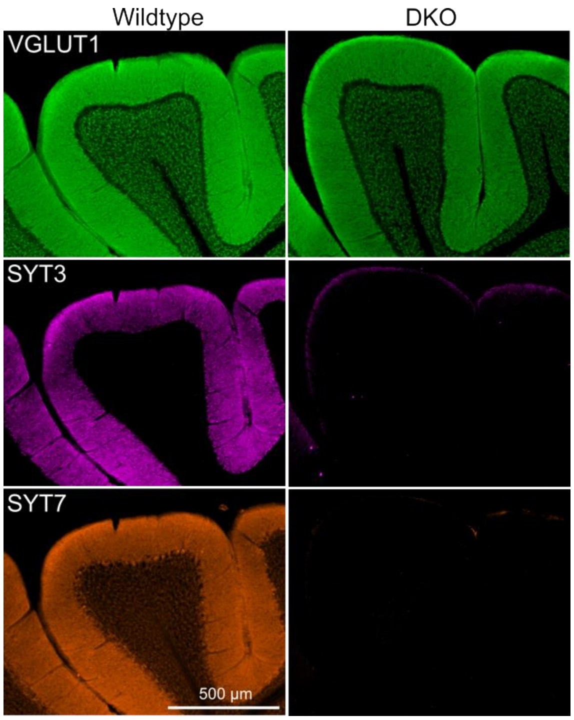

Figure S4 | Immunohistochemistry for SYT3 and SYT7 in the cerebellum, related to Figure 2
Representative low-magnification images showing immunolabeling for VGLUT1, SYT3 and SYT7 in sagittal slices of the cerebellar vermis of WT (left) and SYT3/SYT7 double knockout animals (right). Scale bar: 500 µm.

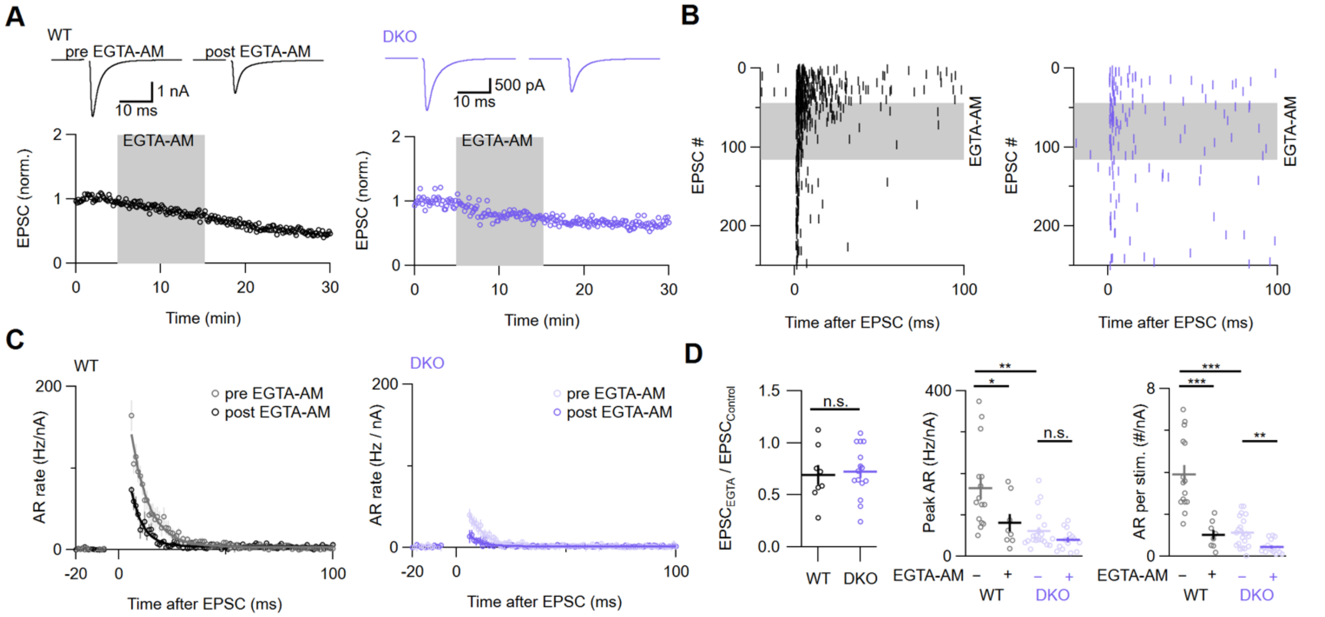

Figure S5 | AR in DKO synapses is sensitive to EGTA-AM, related to Figure 2

A) EPSCs in MLIs elicited by parallel fiber stimulation at 0.125 Hz in WT (black) and DKO (purple) animals before and after a 10 min wash-in and subsequent wash-out of 100 µM EGTA-AM and on top. EPSC amplitudes normalized to the first 5 minutes before wash-in at the bottom.

B) Raster plot of AR from the recordings shown in **A**.

C) Average rates of AR after EGTA-AM wash-on for WT and DKO synapses superimposed with the respective AR in control ACSF (gray) normalized to the EPSC amplitude in control conditions (see Methods).

D) Quantification of EPSC amplitude ratio before and after application of EGTA-AM ($P = 0.79$), the normalized peak AR rate 6-100 ms after an EPSC (WT-control vs. WT-EGTA: $P = 0.02$; DKO-control vs. DKO-EGTA: $P = 0.02$; WT-control vs. DKO-control: $P = 1.57 * 10^{-4}$; Critical α: 0.025), and normalized number of AR events per stimulus before and after treatment with EGTA-AM (WT-control vs. WT-EGTA: $P = 1.58 * 10^{-5}$; DKO-control vs. DKO-EGTA: $P = 9.48 * 10^{-4}$; WT-control vs. DKO-control: $P = 1.92 * 10^{-5}$; Critical α: 0.025).

Averages shown with error bars represent mean ± SEM. Normal distribution was verified with Shapiro-Wilk tests. Subsequently, statistical significances were evaluated using two-tailed Student's t-tests (EPSC-control vs. EPSC-EGTA) and ANOVA followed by two-tailed Student's t-tests (Peak AR and AR per stim). Here, critical thresholds were post-hoc Šidák corrected. The number of experiments is shown in Table S1.

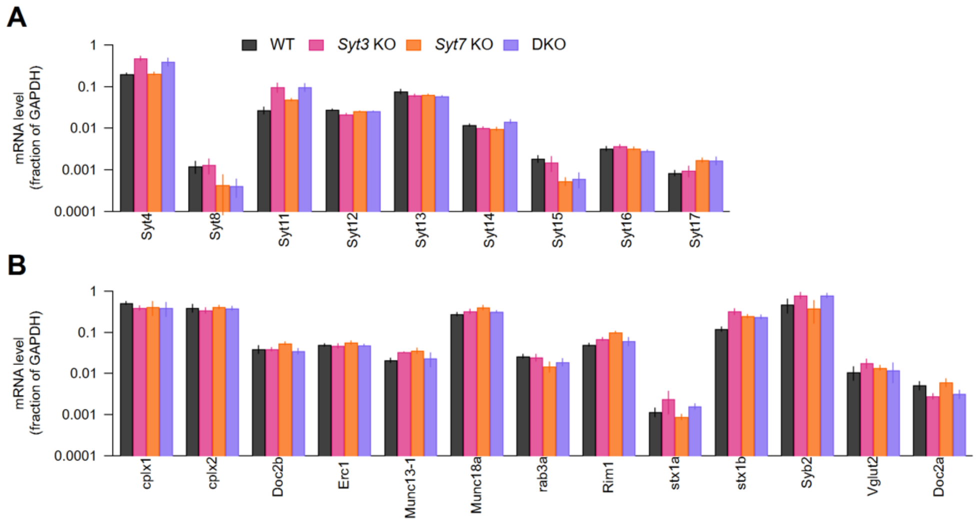

Figure S6 | mRNA expression of Ca^{2+} insensitive synaptotagmins and other synaptic proteins in WT and KO animals, related to Figure 4

mRNA levels measured by RT-PCR for non-Ca^{2+}-binding synaptotagmins (A) and other pre- and postsynaptic genes (B) from cerebellar tissue, normalized to GAPDH. Measurements from cerebellar tissue from 4 mice per genotype with 3 technical replicates per animal. Data represent mean ± SEM.

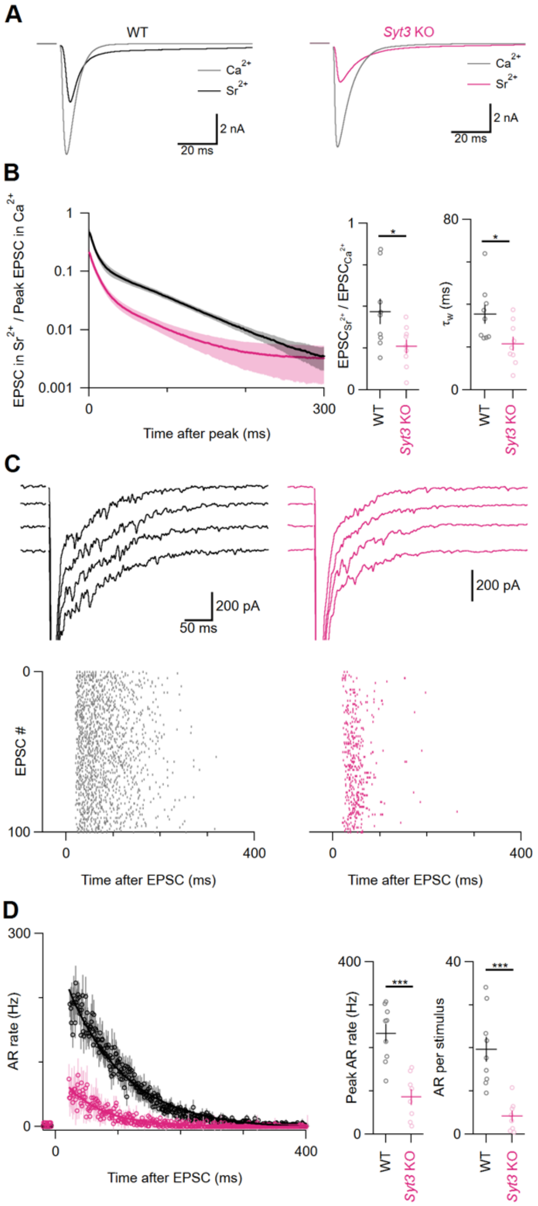

Figure S7 | SYT3 is required for the majority of Sr^{2+}-induced AR from climbing fibers, related to Figure 5

A) Representative average of 100 EPSCs with stimulation artifacts blanked in Purkinje cells elicited by climbing fiber stimulation at 0.125 Hz in Ca^{2+}-containing ACSF, and after wash-on of Sr^{2+}-containing ACSF in WT (black) and SYT3 KO (magenta) animals.

B) Average time course of EPSC decay in Sr^{2+} normalized to the peak EPSC amplitude in Ca^{2+} (left). Ratio of peak EPSC amplitudes in Ca^{2+} and Sr^{2+} (*P* = 0.031), and double-exponential weighted decay time constant of climbing fiber EPSCs (*P* = 0.024) is shown on the right.

C) Example climbing fiber EPSCs in Sr^{2+} (top). Raster plots of AR events for 100 consecutive trials at 0.125 Hz (bottom) (see STAR★Methods).

D) Average rate of AR in Sr^{2+} on the left. Data were fit with double exponentials. Quantification of the peak AR rate 20-100 ms after an EPSC ($P = 1.18 * 10^{-5}$) and average number of AR events per stimulus ($P = 4.69 * 10^{-5}$) on the right.

Averages shown with error bars represent mean ± SEM. Statistical significances were evaluated using Student's t-tests or Kruskal-Wallis tests followed by Dunn's tests. Critical significance thresholds were post-hoc Šidák corrected. Number of experiments is shown in Table S1.

Table S1: Number of recordings, related to STAR Methods

Figure	Synapse/Region	Experiment	Sample	# of recordings	# of animals
Figure 1 D-F	Climbing fiber	EPSC kinetics	WT	10	3
			SYT3 KO	11	3
Figure 1 I+J	Climbing fiber	Asynchronous release	WT	7	3
			SYT3 KO	7	3
Figure 1 K+L	Climbing fiber	Spikelets during CF stimulation	WT	10	3
			SYT3 KO	9	3
Figure 2 E-F	Parallel fiber	EPSC kinetics & Asynchronous release	WT	15	6
			SYT3 KO	19	7
			SYT7 KO	29	9
			DKO	19	6
Figure 2 H	Parallel fiber	Delayed firing in current clamp	WT	12	4
			SYT3 KO	11	3
			SYT7 KO	12	4
			DKO	11	3
Figure 3 D-G	Stratum Oriens interneurons	EPSC kinetics & Asynchronous release	WT	11	3
			SYT3 KO	10	3
			SYT7 KO	10	4
			DKO	8	2
Figure 4 A	Parallel fiber	Ca^{2+}-imaging (Magnesium green)	WT	8	3
			SYT3 KO	13	4
			DKO	5	2
Figure 4 B	Parallel fiber	Ca^{2+}-imaging (Fura-2)	WT	9	3
			SYT3 KO	8	3
			DKO	10	3
Figure 4 C	Parallel fiber	Field recordings	WT	10	3
			SYT3 KO	9	3
			DKO	8	2
Figure 4 D	Cerebellum	RT-PCR	WT	12	4
			SYT3 KO	12	4
			SYT7 KO	12	4
			DKO	12	4
Figure 5 E	Parallel fiber	Asynchronous release in Sr^{2+}	WT	6	3
			SYT3 KO	6	3
			SYT7 KO	8	3
			DKO	8	3
Figure S1 C	Climbing fiber	AR kinetics	WT	8	3
			SYT3 KO	9	4
Figure S1 F	Parallel fiber	Spontaneous EPSC kinetics	WT	33	9
			SYT3 KO	25	10
			SYT7 KO	32	12
			DKO	33	9
Figure S1 I	Stratum Oriens interneurons	Spontaneous EPSC kinetics	WT	11	3
			SYT3 KO	10	3
			SYT7 KO	10	4
			DKO	8	2
Figure S2 A	Purkinje cells	Spontaneous firing rate	WT	9	3
			SYT3 KO	9	2
Figure S2 B	Purkinje cells	Current injection	WT	9	3
			SYT3 KO	8	2

Figure S5	Parallel fiber	EGTA-AM	WT control	15	3
			WT EGTA	8	2
			DKO control	19	6
			DKO EGTA	15	2
Figure S6	Cerebellum	RT-PCR	WT	12	4
			SYT3 KO	12	4
			SYT7 KO	12	4
			DKO	12	4
Figure S7	Climbing fiber	Asynchronous release in Sr^{2+}	WT	9	3
			SYT3 KO	9	3

Table S2: Oligonucleotides used in this study, related to Figures 4 and S6

Gene name	Forward primer sequence	Reverse primer sequence
Vglut1	CCCCCAAATCCTTGCACTTT	AACAAATGGCCACTGAGAAACC
Vglut2	TCCCTCGGACAGATCTAC	CATAGCGGAGCCTTCTTC
Cacna1a	CGACCTCTAAAGACCATCAAGCG	ACGGCTACCACAGCGAAGATGA
Rims1	CAAGGCCTATTGGTGACATCC	CCCACCTTATCATACCACAGC
Munc18a	AGGTGCTACTGGATGAGGACGA	TCGCCAGTGTTCATCCTCTTGC
Munc13-1	GAG CCC AAA GAG TTC AAG	GTC TGG CAT GCT GTC AAT
Gria2	TTCTCCTGTTTTATGGGGACTGA	CCCTACCCGAAATGCACTGTA
SNAP-25	ATCCGCAGGGTAACAAATGATG	CGGAGGTTTCCGATGATGC
Cplx1	AGTTCGTGATGAAACAAGCCC	TCTTCCTCCTTCTTAGCAGCA
Cplx2	GTGTGCTTGAGACAACTGTCACG	GCCATCTGTGACTAACCGTCTC
Dlg4	TCCGGGAGGTGACCCATTC	TTTCCGGCGCATGACGTAG
Rab3a	GAACCTTGCTTCGCCTACTTC	GGGGAGGTAGTCTGCTCTTG
Doc2a	CTGTGATGAGGACAAGCTGAGC	CCGCAGACATTGAAGAGGGTGA
Doc2b	CGACGGCTACGAGTCAGAC	TTCAGGGTGTTCCGAAGAGTT
5'-GAPDH	GTGAACCATGAGAAGTATGACAAC	CATGAGTCCTTCCACGATACC
Center GAPDH	TCAACGACCACTTTGTCAAGC	CCAGGGGTCTTACTCCTTGG
3'-GAPDH	AGTCCCTGCCACACTCAG	TACTTTATTGATGGTACATGACAAGG
Bsn	GGGCAGCCAGAGAACAACTT	GGGACAGAGTAGGGTGACG
Erc1	GTGCTCGATCAGTAGGGAAGG	CCGATGACCTAAGCGAGGG
Stx1a	GAGGAAGGTCTGAACCGCTCAT	CGTTCTCGGTAGTCTGACTGAG
Stx1b	CACTCCACACTCTCACGGAAG	TGCCTCTGGATTCGGTCCTT
Syb2	GCTGGATGACCGTGCAGAT	GATGGCGCAGATCACTCCC
Syt1	CTGTCACCACTGTTGCGAC	GGCAATGGGATTTTATGCAGTTC
Syt2	TCTTGCCTCGTCCCTCGTC	CCACCCAAGCTCCCTTCTT
Syt3	CTCATCTCCTCGAAGCCATAATGT	CTTGGACAACAGCAAAGAGACAGA
Syt4	TGACCCGTACATCAAAATGACAA	GTGGGGATAAGGGATTCCATAGA
Syt5	TGGGCCGGAGTTACATAGATAA	GGCCTAGCTGGTGTTTGTCTG
Syt6	TGGACCAAGTGAGCCTGCTCAT	CGCCAACATCTCATTCCAGTGG
Syt7	AACCCCTCTGCCAACTCCAT	GCGGCTGAGCTTGTCTTTGT
Syt8	GCGGTTTTCAGAACACGAGCCA	GTAGCGTAGTGAGAAGCACAGC
Syt9	CTGCCAAGATTTCATCTACCACC	TCCAAGACACGAAAAGAGAGACA
Syt10	CAGAGAATGTGGACCAGGTCAG	CAGCATCTCGTTCCAATGGTCTC
Syt11	GAGATCACAAATATACGCCCCAG	GCAGCACGTCCACACAAAG
Syt12	GGAGGTGGGTGTCTATGCTG	CTGGTCATTCCAAGGAGGGAC
Syt13	CGGCCCAACAGTTCAACATTA	GCCGGGGACCATAGATGTC
Syt14	TCCAGAGGCGGTTGGATTTTT	TCCGTGTATTGTATCCTGAACCA
Syt15	CAACAAATTCGTCAAGTGCAAGA	CCCAACACCATGTCCCCTG
Syt16	ATGGCGACTGACATCACTCCT	TGGATGCCCTCTTAGGTTTTCA
Syt17	GTCAGAGGTGCTATGAGTCCA	GGGGTCAAAGGAACATCGCT
